# Seaweed-Derived Sulfated Polysaccharides; The New Age Chemopreventives: A Comprehensive Review

**DOI:** 10.3390/cancers15030715

**Published:** 2023-01-24

**Authors:** Prajna Paramita Bhuyan, Rabindra Nayak, Srimanta Patra, Hadi Sajid Abdulabbas, Mrutyunjay Jena, Biswajita Pradhan

**Affiliations:** 1 Department of Botany, Maharaja Sriram Chandra Bhanja Deo University, Baripada 757003, India; 2 Algal Biotechnology and Molecular Systematic Laboratory, Post Graduate Department of Botany, Berhampur University, Bhanja Bihar, Berhampur 760007, India; 3 Cancer and Cell Death Laboratory, Department of Life Science, National Institute of Technology Rourkela, Rourkela 769008, India; 4 Continuous Education Department, Faculty of Dentistry, University of Al-Ameed, Karbala 56001, Iraq; 5 School of Biological Sciences, AIPH University, Bhubaneswar 752101, India

**Keywords:** apoptosis, cancer, chemoprevention, sulfated polysaccharides

## Abstract

**Simple Summary:**

Sulfated polysaccharides are powerful chemotherapeutic or chemopreventive agents that have anti-cancer properties by increasing immunity and driving apoptosis in several cancer cell lines. Sulfated polysaccharides have significant antioxidant and immunomodulatory potentials, which contribute to their disease-preventive effectiveness with low cytotoxicity and good efficacy therapeutic outcomes in cancer via dynamic apoptosis modulation. Furthermore, it can be used as a dietary supplement or as an adjuvant treatment for cancer.

**Abstract:**

Seaweed-derived bioactive compounds are regularly employed to treat human diseases. Sulfated polysaccharides are potent chemotherapeutic or chemopreventive medications since it has been discovered. They have exhibited anti-cancer properties by enhancing immunity and driving apoptosis. Through dynamic modulation of critical intracellular signalling pathways, such as control of ROS generation and preservation of essential cell survival and death processes, sulfated polysaccharides’ antioxidant and immunomodulatory potentials contribute to their disease-preventive effectiveness. Sulfated polysaccharides provide low cytotoxicity and good efficacy therapeutic outcomes via dynamic modulation of apoptosis in cancer. Understanding how sulfated polysaccharides affect human cancer cells and their molecular involvement in cell death pathways will showcase a new way of chemoprevention. In this review, the significance of apoptosis and autophagy-modulating sulfated polysaccharides has been emphasized, as well as the future direction of enhanced nano-formulation for greater clinical efficacy. Moreover, this review focuses on the recent findings about the possible mechanisms of chemotherapeutic use of sulfated polysaccharides, their potential as anti-cancer drugs, and proposed mechanisms of action to drive apoptosis in diverse malignancies. Because of their unique physicochemical and biological properties, sulfated polysaccharides are ideal for their bioactive ingredients, which can improve function and application in disease. However, there is a gap in the literature regarding the physicochemical properties and functionalities of sulfated polysaccharides and the use of sulfated polysaccharide-based delivery systems in functional cancer. Furthermore, the preclinical and clinical trials will reveal the drug’s efficacy in cancer.

## 1. Introduction

The current global population explosion and altered dietary and lifestyle practices are considered critical factors for disease occurrence. Numerous infection-driven diseases, along with Alzheimer’s, Parkinson’s, diabetes, cancer, and other neurological disorders, pose a severe risk to the human lifespan [1]. Cancer, a collection of numerous pathological problems brought on by unchecked cell growth, has detrimental effects on individual health care [2]. According to estimates from 2019, there are more than 200 deadly cancer kinds that cause more than 9.6 million deaths annually worldwide [3]. The leading causes of death are skin, stomach cancer, breast, lung, prostate, and colorectal cancer [4]. Instances of mortality in low- and middle-income nations are documented in about 70% of cases [5]. Based on epidemiological studies, the World Health Organization (WHO) predicted 9.6 million cancer-related deaths and 18 million new cases in 2018 [6]. Several homeostatic systems are disrupted by the uncontrolled proliferation of cancer cells leading to their invasiveness and metastasis owing to genetic changes [7]. Cancer treatment methods include surgery, chemotherapy, radiation therapy, and immunotherapy; chemotherapy is the most frequently used [7,8,9]. Chemotherapy is a common and efficient cancer treatment that damages several important organs by causing cytotoxicity in both cancerous and non-cancerous cells [10]. Drug tolerance is the main concern in cancer treatment in order to eliminate side effects and severe reactions [11,12]. Therefore, it is crucial to develop and look for anti-cancer drugs with fewer side effects and higher tolerance.

Chemotherapy sometimes creates an unfavourable setting and irreversible organ damage surrounding the target site. Additionally, cellular lenience to the medications possesses additional therapeutic difficulties. Therefore, it is desired to find fresh therapeutic agents with low side effects to abide all the adverse conditions [13]. The naturally occurring bioactive compounds used as drugs have a variety of therapeutic applications [14,15,16,17,18]. Additionally, most medicinal drugs are either natural compounds or synthetic equivalents [19]. Due to their varied chemical makeup and bioavailability, marine natural products (MNPs) have recently been investigated for their potential as therapeutic candidates [20,21,22,23,24,25].

Algal biodiversity is abundant in marine and freshwater settings and contributes to the main bioactive metabolites [24,26,27,28,29,30,31,32,33,34,35,36]. Seaweeds are found in both freshwater and saltwater, and they play a significant part in preserving the ecology and biodiversity of marine ecosystems [37,38]. Anticoagulant, anticancer, antidiabetic, antiviral, immunomodulatory, antiangiogenic, anti-inflammatory, antiadhesive, and anti-neurodegenerative properties of sulfated polysaccharide use as potential therapeutic agents [39,40,41,42,43,44]. Fucoidan, porphyran, carrageenan, and ulvan are sulfated polysaccharides often extracted from brown, red, and green algae and contain sulfate groups that have the possibility as therapeutic diligences against many malignancies [45,46,47]. Sulfated polysaccharides are expected to be used as chemotherapeutic pharmacological agents in clinical practice due to their enormous structural variety and robust antioxidant capacity [48]. Additionally, its high absorption, cheap maintenance costs, improved production, and use as food supplements make it a more sought-after chemotherapy drug [49].

Previous reports have discussed the anticancer properties of algal-derived sulphated polysaccharides [50,51,52]. However, the exact mechanism is not fully understood or discussed. Moreover, the context-specific drug targets, cancer subtypes and tumour microenvironment are not discussed [52,53,54]. The present form of the manuscript discusses the mechanistic involvement of these algal-derived sulphated polysaccharides in the induction of cell death pathways. The multi-target specific one-drug therapy has also been discussed keeping the tumour heterogeneity in mind.

The systematic analysis of the origin and mechanistic overview of the sulfated polysaccharide regulatory pathways used in cancer prevention are the main topics of this review. To comprehend therapeutic intervention in the context of cancer prevention, we have also concentrated on the chemical complexity and sources of sulfated polysaccharides. Future clinical and nano drug delivery uses are suggested by this review, which also takes into account the potential function of sulfated polysaccharides in cancer prevention.

## 2. Intricate Role of Apoptosis in Cancer Treatment: The Programmed Cell Death

**Figure 1:**
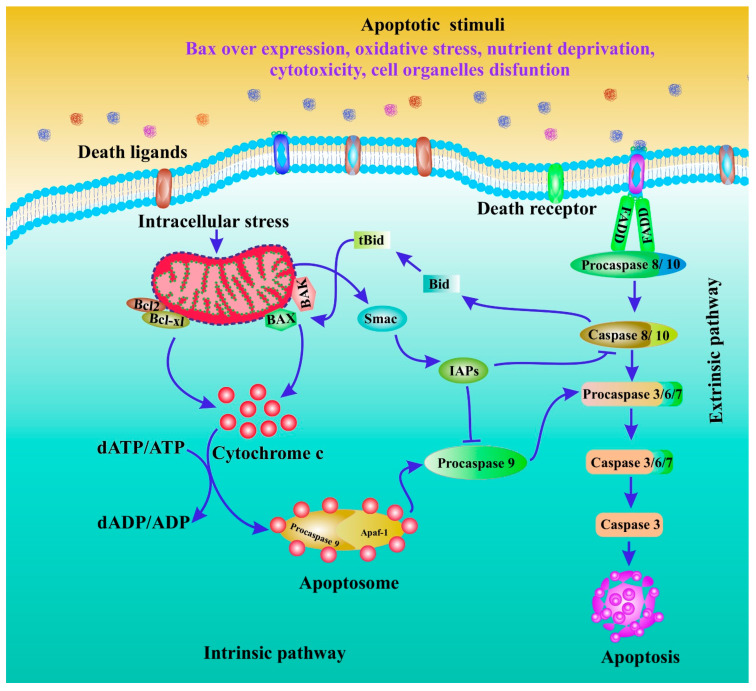
Role of apoptosis in cancer treatment.

Understanding the pathogenesis of diseases brought on by apoptosis dysfunction requires an understanding of the mechanisms of apoptosis. The creation of medications that specifically target apoptotic genes or pathways may benefit from this. Because they function as both initiators and executors, caspases are essential parts of the apoptosis mechanism. There are three distinct pathways by which caspases can be activated. Intrinsic (or mitochondrial) and extrinsic are the two apoptosis initiation pathways that are most frequently discussed (or death receptor) (Figure 1). Both pathways ultimately lead to the execution stage of apoptosis, which is a common pathway. The intrinsic endoplasmic reticulum pathway is a third, less well-known initiation pathway [55].

Numerous caspases are activated during the apoptosis execution stage. Caspase 9 mediates the intrinsic pathway, while Caspase 8 mediates the extrinsic pathway. Both intrinsic and extrinsic pathways converge on caspase 3 to complete apoptosis. Nuclear apoptosis is caused by the degradation of the caspase-activated deoxyribonuclease inhibitor by caspase 3 [56]. Additionally, protein kinases, cytoskeletal proteins, DNA repair proteins, and endonuclease inhibitory subunits are cleaved by downstream caspases. Additionally, they influence the cytoskeleton, cell cycle, and signalling pathways, all of which help to shape the specific morphological changes that take place during apoptosis [56].

Apoptosis is an energy-dependent programmed cell death characterised by membrane blebbing, shrinking cytoplasmic chromatin condensation, and nuclear disintegration. Apoptosis is the process by which cells die without causing inflammation [57,58]. Additionally, it can be started by mitochondrial-mediated mechanisms or surface death receptors (DR; extrinsic apoptosis) (intrinsic apoptosis) [59,60,61,62]. Both pathways cause executive caspases to be activated, which cleave molecules related to the structural and regulatory molecules of the network of apoptotic cells [46,63,64]. After pathogenic stressors, apoptosis is a cell death mechanism that aids in maintaining cellular homeostasis [65]. Malignant cells typically go through a series of genetic mutations to survive pathogenic stimuli. Apoptosis resistance or decreased apoptosis promotes carcinogenesis [66,67,68]. Cancer cells frequently avoid apoptosis by rebalancing the pro- and anti-apoptotic protein balance. Cancer cells can also avoid apoptosis if their caspase activity is low and their DR signalling is compromised [62,69,70]. Bcl-2 family proteins, an inhibitor of apoptosis proteins (IAPs), p53, executioner caspases, and DRs are frequently affected by cancer cells. These molecular genes and their related pathways are critical in cancer therapies because they cause apoptotic cell death [71,72,73,74,75]. Apoptosis’s typical role in cancer treatment is depicted (Figure 1).

## 3. Seaweeds: The Chief Contributor of Sulfated Polysaccharides

Due to their enormous biodiversity and use as food and traditional medicine worldwide, seaweeds are thought to be a good source of bioactive chemicals [76,77,78]. The therapeutic effects of a number of seaweed-derived bioactive chemicals, their unprocessed extracts, and partially purified polysaccharides on a range of human diseases have been investigated [54,79,80]. Their antioxidant qualities aid the ability of the phytoproducts made from seaweed to resist disease. Brown seaweeds have various physical and functionally distinct polysaccharides, including alginic acids, and fucoidans [81,82]. In biotechnology, medicine, and food preparation, sulfated polysaccharides are frequently used [83,84]. Polyphenols, free amino acids, iodine-containing substances, vitamins, and lipids isolated from seaweeds are examples of low molecular metabolites used in food processing and medicine [81,85]. The sulfated polysaccharides are physiologically active, highly branched, different from monosaccharide composition, and have a higher molecular weight. Long chains of linked sugar molecules make up the fucoidan, which is decorated with sulfate groups [86]. Sulfated polysaccharides’ ability to fight off many malignancies is mainly attributed to their antioxidant capability [87,88,89]. It is well known that the sulfated polysaccharides derived from seaweeds are effective anticancer drugs. 

Seaweed contains a variety of sulfated polysaccharides. According to their chemical makeup, polysaccharides are categorised as galactans, and sulfated xylans, sulfuric acid polysaccharides (generally found in green algae). Brown algae also contain fucoidan [90]. Red algae frequently contain agar, carrageenans, xylans, and floridean. Several algal sulfated polysaccharides could be used as therapeutic candidates to address a variety of human health inequalities [91]. Sulfated galactans known as carrageenans are frequently used in the food and drug industries. soluble fibres, like fucans, are found in brown seaweeds. On the other hand, red seaweeds are abundant in soluble fibres like xylans, floridean starch, and sulfated galactans (agars and carrageenans) [92]. Green algae also contain xylose, galactose, uronic acids, arabinose, and rhamnose, as well as mannans, xylans, starch, and polysaccharides with ionic sulphate groups. There are many types of soluble and insoluble fibres in polysaccharides [93,94]. Compared to their dry weight, seaweeds provide a more significant percentage of dietary fibres (between 25% and 75%) than those found in fruits and vegetables [95]. Consuming dietary fibre from algae has many positive health effects since it acts as an antitumor, anticancer, anticoagulant, and antiviral agent. In brown macroalgae, sulfated polysaccharides are extensively distributed in the cell walls [80]. Among other biological actions, sulfated polysaccharides act as an antioxidant, anti-inflammatory, anticoagulant, anticancer, antiviral, antidiabetic, and antithrombotic agent. They also alter how the human immune system [7]. Additionally, fucoidan, which is rich in brown seaweeds and is the second largest source of sulfated polysaccharide, promotes intestine metabolism in human health [94].

### The Structural Complexity of Seaweed-Derived Sulfated Polysaccharides

Research is increasingly focusing on polysaccharides, which are found in seaweeds and have anticancer, antioxidant, anti-coagulant, and anti-inflammatory properties [7,96]. Polysaccharides are large molecules classified by a monomeric unit as homopolysaccharides, homoglycans, heteropolysaccharides, or heteroglycans. Polysaccharides are also classified according to their seaweed origin as brown, red, green, or blue. Fucoidan (a sulfated polysaccharide), are the main component of brown seaweed. Agars, xylans, carrageenans, floridean starch (glucan that resembles amylopectin), water-soluble sulfated galactans, and porphyran are some products made from red algae. Green seaweeds contain sulfated galactans, xylans, and polysaccharides. Seaweed contains a variety of polysaccharides, with some genera—including *Ascophyllum*, *Porphyra*, and *Palmaria*—containing up to 76% polysaccharide by dry weight [97].

## 4. Disease Preventive Activity of Sulfated Polysaccharides: The Magic Bullets

Numerous studies have demonstrated that the biological activity of polysaccharides is influenced by their molecular weight, conformational state, chemical composition, and glycosidic connections [98]. Understanding the relationship between molecular weight and essential properties, such as polysaccharide viscosity, conformation, water solubility, and others, is important in cancer [99,100]. Porphyran’s with a lower molecular weight (LMW) have more potent antioxidant properties [101,102]. Since porphyran has a lower mean molecular mass, it has a more significant ROS-scavenging activity [103]. Additionally, the byproduct of porphyran acid hydrolysis, oligo-porphyran, has the potential to both prevent and treat a number of cancers. A higher irradiation exposure dose and porphyran with a lower molecular weight were required because gamma radiation damaged the anti-cancer response of porphyran derived from *P. yezoensis* [45]. Contrary to earlier research that claimed lower molecular weight porphyran has more potent anti-cancer activity, porphyran inhibited cancer cell lines HeLa and Hep3B more potently than the degraded products. The composition of the monosaccharide or sulphate did not change significantly [100]. Therefore, future research should focus on how the molecular weight of porphyran and their anti-cancer activity are related.

In Asian nations like Japan, China, Thailand, and South Korea, edible seaweeds are valued as a wholesome food source. The fight against cancer necessitates the use of polyphenols, terpenes, phycobiliproteins, carotenoids, phlorotannins, pigments, and polysaccharides [104]. Antioxidants found in seaweed’s anti-cancer properties help stop the spread of cancer. As cancer progresses, antioxidants are crucial because they inhibit tumour growth without causing cytotoxicity [105]. For instance, a mouse model of sarcoma 180 was successfully treated with an immune-stimulating sulfated polysaccharide from *Champia feldmannii* without cytotoxicity [106]. The polysaccharides from the *Gracilaria lemaneiformis* induced splenocyte proliferation, macrophage phagocytosis, and tumour inhibition. Mice with H22 hepatoma cell transplants had higher levels of IL-2 and CD8+ T lymphocytes in their blood [107]. A sulfated polysaccharide from *C. feldmannii* showed anti-cancer efficacy in Swiss mice in vitro and in vivo. Increasing the production of OVA-specific antibodies improves immunity [106]. Fucoidans’ anti-cancer properties have been confirmed in a diversity of cancers, including stomach, breast, lung, and liver cancers [7]. Fucoidan has received more attention than porphyran and other sulfated polysaccharides. Sulfated polysaccharides from green, brown, and red algae have sparked a lot of interest in this context due to their anticancer properties.

The physicochemical characteristics of the different sulfated polysaccharides and their wide range of therapeutic potential (Table 1) will be leading this research. Some physicochemical properties of sulfated polysaccharides have been reported, including ionic solubility, crosslinking, biocompatibility, nontoxicity, rheological properties, and biodegradability [108,109,110]. These properties are important characteristics of sulfated polysaccharides that have sparked a lot of interest in their application. Fucoidan’s primary properties are ionic crosslinking and solubility [109]. The water-soluble sulfated polysaccharides facilitate the development of fucoidan and other positively charged molecule-based delivery systems. Fucoidan’s negatively charged sulphate groups, for example, could be communal with chitosan’s ammonium groups to form nanoparticles, hydrogels, and comestible films for nutraceutical delivery [111,112].

Nontoxicity is an important property in addition to ionic crosslinking and solubility. Except as previously stated, biodegradability and biocompatibility are critical factors in facilitating the use of sulfated polysaccharides in therapeutic and drug delivery systems. Researchers have recently become interested in sulfated polysaccharides because of their excellent biocompatibility and biodegradability. The biodegradability of sulfated polysaccharides can increase the bioavailability and delivery effectiveness of bioactive ingredients. Depolymerization and purification can generally increase the biodegradability of sulfated polysaccharides by lowering their molecular weight, but this method is too expensive to be widely used [113].

### Apoptosis Modulatory Seaweeds Derived Sulfated Polysaccharides

As anticancer anti-angiogenic, and anti-inflammatory drugs, sulfated polysaccharides have a variety of biological effects [43,114]. Sulfated polysaccharides isolated from different marine habitats have been extensively studied and found to be effective anticancer mediators against various cancer cell lines by modulating numerous cell survival pathways and inducing apoptosis [43,46]. Therefore, Sulfated polysaccharides secluded from various green, brown, and red seaweeds from various marine habitats have been extensively studied for their ability to dynamically regulate cell death pathways. These polysaccharides are effective anticancer agents because they modulate numerous cell survival pathways and induce apoptosis. Sulfated polysaccharides are widely used in cancer therapies as well as precision medicine to develop next-generation drugs.

**Green seaweeds:** Green algae, also known as Chlorophyta, are an influential group of marine algae that are a source of polysaccharides [115]. However, green algae cell wall polysaccharides have received less attention than red (agarans and carrageenan) and brown algal polysaccharides (fucoidan) [96]. Nonetheless, the study of sulfated polysaccharides derived from green seaweeds has piqued the scientific community’s interest in recent years, primarily because of their structural diversity, biological, and physicochemical properties [116]. Furthermore, due to their variety of glycosidic linkages that result in branched structures and attached sulphate groups with various special distributions, sulfated polysaccharides are the most bioactive and promising candidates [117].

**Figure 2:**
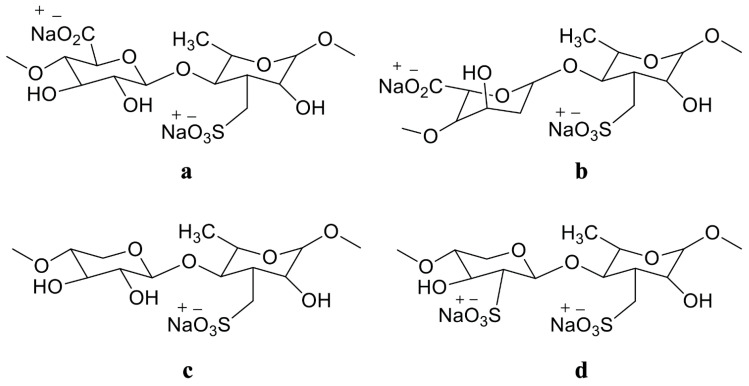
The Molecular structure of ulvan is drawn in Chemdraw 12.0 Ultra. Nomenclature and structure of the major repeating disaccharide units that comprise ulvan. Ulvanobiuronic acid A3s contains glucuronic acid attached to rhamnose 3-sulfate (**a**); while the similar B3s also contains rhamnose 3-sulfate but has iduronic acid (**b**) in the place of glucuronic acid. Ulvanobioses are comprised of rhamnose 3-sulfate attached to xylose (**c**); Xylose can contain a sulfate group, as seen in U2′s,3s (**d**).

Ulvan are sulfated polysaccharides that are water-soluble and derived from the cell walls of green algae. They are present in plants belonging to the genera Ulva, Enteromorpha, Monostroma, Caulerpa, and others. They consist of repeating disaccharide moieties like sulfated rhamnose and uronic acid (glucuronic or iduronic). Glycosaminoglycans, which are present in the extracellular matrix of animal connective tissues, have a structure that is comparable to that of ulvan disaccharide moieties. Some ulvan also have xylose residues visible (Figure 2) [118]. Highly pyruvate 1,3-D-galactan sulphate from the *Codium yezoense* and a polysaccharide similar to it from *Codium isthmocladium* are two other types of polysaccharides found in green algae [119,120]. Sulfated β-D-mannans have also been discovered, such as those isolated from *Codium vermilara* [121]. The molecular structure of ulvan sulfated polysaccharide is displayed in Figure 2.

Sulfated polysaccharides isolated from various tropical green algae have recently been found to have antioxidant and antiproliferative properties. After 72 h of incubation, HeLa cell proliferation was reduced by 36.3% to 58.4% by the polysaccharide isolated from *Caulerpa prolifera* [122]. Two polysaccharide fractions from the *Caulerpa racemosa*, a green alga, showed antitumor activity at a dose of 100 mg/kg/day, with inhibition rates of H22 tumour transplanted in mice of 59.5–83.8% (48 h) and 53.9% (14 days), respectively [123].

Through in vivo and in vitro experiments, water-soluble sulfated polysaccharide fractions of *Enteromorpha prolifera* were found to stimulate immunity. These polysaccharides significantly increased ConA-induced splenocyte proliferation and cytokine production through elevated m-RNA expression [124]. Ulvan from *Ulva rigida* stimulated the secretion and activity of murine macrophages, increased COX-2 and NOS-2 expression, and more than doubled the expression of some cytokines [125]. Ulvans from *Ulva pertusa* stimulated nitric oxide and cytokine production while causing little cytotoxicity against tumour cells [126]. Several studies on the antioxidant activity of ulvan in experimental D-galactosamine-induced hepatitis in rats have been published [127,128]. Polysaccharides derived from green algae have potent immunomodulatory and antioxidant properties, implying that they could be used to prevent cancer.

Ulvan’s anticancer activity has recently been discovered in U. australis, U. lactuca, U. ohnoi, and *U. rigita* [129]. Several studies have investigated ulvan in toxicity and cell viability to test its anticancer activity, specifically for anti-breast cancer, anti-colon, and anti-cervical cancer activity [129,130,131,132]. Ulvan contains sulfated polysaccharides, which inhibit the proliferation of hepatocellular carcinoma and induce apoptosis. By lowering oxidative stress, sulfated polysaccharides protect the liver from DNEA-induced damage [133]. Additionally, they enhance apoptosis, reduce oxidative stress and inflammation, and strengthen the antioxidant defence system in DMBA-treated mice [130]. Ulvan was less toxic to A459 and LS174 cells (IC_50_ > 200 mg/mL), but it was more effective against Fem-x and K562 cells (IC_50_ 74.73 and 82.24 mg/mL, respectively) when it came to preventing moderate cytotoxicity [134]. With IC_50_ values ranging from 21 to 99 µg/mL, ulvan reduced tumour growth in MCF-7 and HCT-116 cells [132] and strong ligand bonds appear to connect this to sulfated polysaccharides [135]. Ulvan inhibited the growth of hepatocellular carcinoma (IC_50_ 29.67 ± 2.87 µg/mL), human breast cancer (IC_50_ 25.09 ± 1.36 µg/mL), and cervical cancer (IC_50_ 36.33 ± 3.84 µg/mL) [131]. However, Caco-2 cell proliferation or differentiation can be inhibited by low molecular weight polysaccharides (5000 Da), usually oligosaccharides [129].

Sulfated polysaccharides have an antiproliferative effect, but it depends on the cell type. Sulfated polysaccharide TPs (precipitated in alcohol) extracted from the green alga Codium bernabei exhibited low cytotoxicity on HCT-116 and MCF-7 cell lines in comparison to APs (precipitated in acid media). On the other hand, the HL-60 cell lines showed little cytotoxicity when exposed to the APs [51]. Due to its strong antioxidant activity, *Enteromorpha* spp. extract has antiproliferative effects on cancer cell lines like *Fem-x*, *A549, LS174, and K562* [136]. Additionally, a different solvent extract of *Enteromorpha compressa* extract induces anticancer activity via apoptosis in oral cancer cell lines Cal33 and FaDu [38].

**Figure 3:**
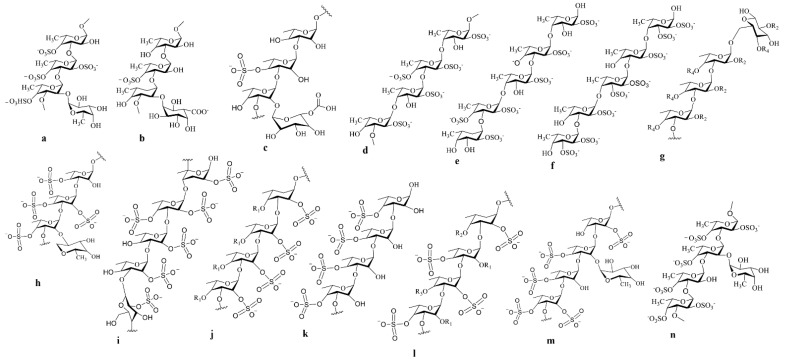
The Molecular structure of different types of sulfated polysaccharides such as fucoidan, with potential therapeutic effects, are drawn in Chemdraw 12.0 Ultra. The different fucoidan structures derived from different brown seaweeds such as Laminaria saccharina (**a**); *Cladosiphon okamuranus* (**b**); *Cladosiphon okamuranus* (**c**); *Fucas evanescens* (**d**); *Fucas serratus* (**e**); *Ascophyllum nodosum/ F. vesiculosus* (**f**); *Sargassum mcclurei* (**g**); *Laminaria saccharina* (**h**); *Turbinaria conoides* (**i**); *Sargassum cichorioides* (**j**); *Turbinaria ornata* (**k**); *Undaria pinnatifida* (**l**); *Chorda filum* (**m**); and *Chorda filum* (**n**).

**Brown seaweeds:** Brown seaweeds are the most promising sources of sulfated polysaccharide and displayed the most promising anticancer activity against various cancer cell lines. The typical sulfated polysaccharide structure derived from brown seaweeds is displayed (Figure 3). Lewis lung cancer cells (LCC) and melanoma B16 cells were discovered to be sensitive to the fucoidan isolated from *Sargassum* sp. [137]. It reduced cell proliferation and dose-dependently promoted apoptosis, as shown by morphological alterations. The fucoidans from *S. hemiphyllum* inhibited the growth of breast cancer by upregulation of miR-29c and downregulation of miR-17-5p. Furthermore, it was clear that after fucoidan administration, EMT progression was slowed by amplified E-cadherin and reduced N-cadherin expression. Furthermore, activation of the pathway of phosphoinositide 3-kinase/Akt has promoted apoptosis in breast cancer cells [138]. Fucoidan from *L. gurjanovae* demonstrated an anti-neoplastic effect in rat epidermal JB6 Cl41 cells by delaying EGFR phosphorylation. It controlled EGF-induced c-jun signalling and inhibited the action of activator protein-1 (AP-1) [139].

Fucoidan derived from *F. vesiculosus* inhibited cell proliferation and arrested the cell cycle in ovarian cancer (ES2 and OV90) cells. It also produced ROS, which regulated intrinsic apoptosis. By suppressing the PI3K and MAPK signalling pathways, ER stress also promoted apoptosis. It also demonstrated anticancer effects on human mucoepidermoid carcinoma by modifying the p-38 MAPK, ERK1/2, and JNK pathways (MC3) [140]. Further, it reduced the amount of calcium in the cytosol and mitochondria to support apoptotic cell death. Similar extraction techniques for fucoidan produced from *F. vesiculosus* showed in vivo anticancer efficacy in the zebrafish xenograft and fli1 Tg model [141]. In HepG2 and HeLa G-63 cells, fucoidan from *Fucus vesiculosus* demonstrated potent anticancer activity. Fucoidan was discovered to be more effective in human liver cancer cells (HepG2) [142]. Fucoidan from *Fucus vesiculosus* increased MMP, which induced caspase-3-dependent apoptosis in human burkitt’s lymphoma (HS-Sultan) cells. Furthermore, reports of caspase-independent apoptotic cell death in HS-Sultan cells were seen after fucoidan administration. Additionally, fucoidan prevented the ERK and GSK pathways from being phosphorylated, both of which were necessary for the activation of apoptosis [143]. Its low IC_50_ (34 µg/mL) activated pro-caspase-3, pro-caspase-9, and caspase-3/7 while downregulating Bcl-2 in HCT-15 cells [144]. The ability of anti-apoptotic proteins like Bcl-xl, Bcl-2, and Mcl-1 to cause apoptosis in MDA-MB231 cells was inhibited by fucoidan at IC_50_ (820 µg/mL) [46]. Fucoidan (IC_50_; 20 µg/mL) therapy led to a similar fluctuation in the expression of Bad, Bcl-2, Bim, Bcl-xl, and Bik in colon cancer cell lines [144].

Fucoidan derived from *C. okamuranus* was combined with Con A, and it promoted intrinsic apoptosis by caspase-3/7 induction in HL60 cells [145]. In addition, glutathione depletion and NO production were significant mediators of apoptosis in human leukaemia cells, as were the activation of MEKK1, ERK1/2, MEK1, and JNK [146]. Fucoidan from C. novaecaledoniae was extracted and used to induce intrinsic apoptosis in HeLa, MCF-7, MDA- MB-231, and HT1080 cells. This intrinsic apoptosis was accompanied by MMP, DNA fragmentation, nuclear condensation, and phosphatidylserine externalisation [147]. *C. okamuranus* fucoidan induced caspase-dependent apoptosis in U937 cells by inducing the caspase-3 and -7 pathways [148]. Additionally, it enhanced mouse cell-mediated immunity, phagocytes, and immune cell proliferation in the in vivo model [149]. Additionally, in normal stomach (Hs 677.St) cells, fucoidan isolated from *C. okamuranus* reduced cellular damage brought on by 5-fluorouracil (5- FU) [150]. In this setting, significant anti-proliferative activity in MCF-7 cells was observed, with no cytotoxicity to human mammary epithelial cells. There was an increase in caspase-7, caspase-8, and caspase-9 activity, internucleosomal DNA fragmentation, and chromatin condensation in both cell lines [151]. Fucoidan therapy has also been reported for caspase-independent cell death in MCF-7 [152]. Hydrolyses increase the luminal fucoidan content, which is a potent chemopreventive mediator of colon cancer, because they do not digest these fucoidans [153]. Fucoidan (0–20 µg/mL) therapy promoted mitochondrial death in HT-29 and HCT116 cells via caspase-3 regulation. Extrinsic apoptosis in HT-29 cells has also been reported recently [144]. The anticancer properties of *C. okamuranus* low molecular weight fucoidan (LMWF; 6.5–40 kDa), high molecular weight fucoidan (HMWF; 300–330 kDa), and intermediate molecular weight fucoidan (IMWF; 110–138 kDa) were demonstrated in a colon carcinoma tumour-bearing rat model [154]. Fucoidan (MW 5100 kDa) from U. pinnatifida induced apoptosis in human prostate cancer (PC-3) cells via induction of ERK1/2 MAPK, inhibition of p38 MAPK, and PI3K/Akt pathway. Furthermore, the downregulation of the Wnt/-catenin pathway aided the progress of apoptosis [155]. Fucoidan also amplified the p21Cip1/Waf pathways in PC-3 cells. Furthermore, it reduced E2F-1 cell cycle-related proteins while increasing Wnt/-catenin pathways. GSK-3 activation reduced the expression of c-MYC, and cyclin D1, which aided anti-proliferative activity [156]. These fucoidans were found to have anticancer activity in HeLa, A549, and HepG2 cells by altering the previously mentioned critical cellular signalling pathways [157].

Fucoidan from *F. vesiculosus* induced apoptosis in cancer cell lines including NB4, THP-1, and HL-60. Fucoidan administration activated caspases-3, -8, and -9, cleaved Bid, and altered MMP in HL-60 cells. The initiation of apoptosis had a comparable effect in U937 cells. Moreover, in U937 cells, fucoidan therapy increased MMP (mitochondrial membrane potential) and cytosolic cytochrome C release, as well as the Bax/Bcl-2 ratio. Caspase inhibitors, on the other hand, delayed the onset of apoptosis, demonstrating that fucoidan-regulated caspase activity was accountable for apoptosis induction. Furthermore, treatment with SB203580, a specific p38 MAPK inhibitor, was accountable for apoptosis discount, demonstrating the importance of MAPK in activating apoptosis [158]. Fucoidan therapy inhibited the G1 cell cycle in EJ cells by affecting cyclin D1, cyclin E, and Cdks (cyclin-dependent kinases). Furthermore, it inhibits Rb phosphorylation, which results in cellular ageing [159].

Fucoidan from *F. vesiculosus* inhibited the growth of MCF-7 cells by stopping the cell cycle at the G1 phase and lowering CDK-4 and cyclin D1 levels. Furthermore, by cleaving PARP and Bid, decreasing Bcl-2 and increasing Bax, it induced ROS-dependent apoptosis. MCF-7 cells exhibited the onset of intrinsic apoptosis via regulation of caspase-7, -8, and -9 and cytosolic cytochrome C release [160,161]. Furthermore, fucoidan from *F. vesiculosus* therapy reduced cell migration and invasion as well as EMT in MCF-7 cells by downregulating MMP-9 and overexpressing E-cadherin [162]. Fucoidans derived from *F. vesiculosus* inhibited growth in MDA-MB-231 and 4T1xenograft female Balb/c mouse cells, ensuing in less metastatic lung nodule development. The effective setback of TGFR-induced EMT was achieved mechanistically by downregulating TGFRII and TGFRI. The cases mentioned above have all been associated with the upregulation of epithelial markers and their phosphorylation of Smad2/3 Smad4 expression, phosphorylation of Smad2/3 Smad4 expression, and downstream signalling molecules [163]. Furthermore, caspase-3 activation, cytosolic cytochrome C release, downregulation of Bcl-2, and increased Bax expression induced apoptosis. In addition, the regulation of VEGF, Survivin, and ERKs expression aided in the commencement of apoptosis [164].

In MDS/AML and SKM1 cell line, treatment with marketed fucoidan (100 µg/mL for 48 h) caused cell cycle arrest (G1 phase) and Fas instigation to induce extrinsic apoptosis via caspase 8 and 9 modulations. Furthermore, it influenced PI3K/Akt pathway in a ROS-dependent manner, thereby promoting apoptosis [165]. It altered p-Akt, p-PI3K, p-P38 and p-ERK, to modulate MAPK and PI3K/Akt signalling pathways in DU-145 cells (prostate cancer). Furthermore, it increased Bax expression while decreasing Bcl-2, PARP cleavage, and caspase-9 expression in a concentration-dependent manner [166]. Fucoidan administration induced apoptosis in osteosarcoma (MG-63) cells (evidenced by cellular blabbing, nuclear disintegration, and chromatin condensation) [167].

Treatment with marketed synthetic fucoidan increased ROS-regulated apoptosis in human bladder cancer (5637) cells by activating mitochondrial membrane potential (MMP), increasing the Bax/Bcl-2 ratio, and increasing cytosolic cytochrome C release. Furthermore, inhibition of PI3K/Akt signalling and anti-telomerase activities promoted apoptotic cell death in 5637 human bladder cancer cells via downregulating telomerase Activity [168]. Furthermore, AKT signalling activation was claimed to be critical in inhibiting proliferation and suppressing bladder cancer cells’ ability to migrate and invade [169]. Fucoidan inhibited the cell cycle in 5637 and T-24 cells (human bladder carcinoma) by altering the expression of p21/WAF1, cyclins, and CDK. Furthermore, MMP-9 inhibition via AP-1 and NF-kB reduced bladder cancer cell proliferation [169]. Sulfated polysaccharides from brown algae as potent anticancer agents are displayed in Table 2.

**Figure 4:**
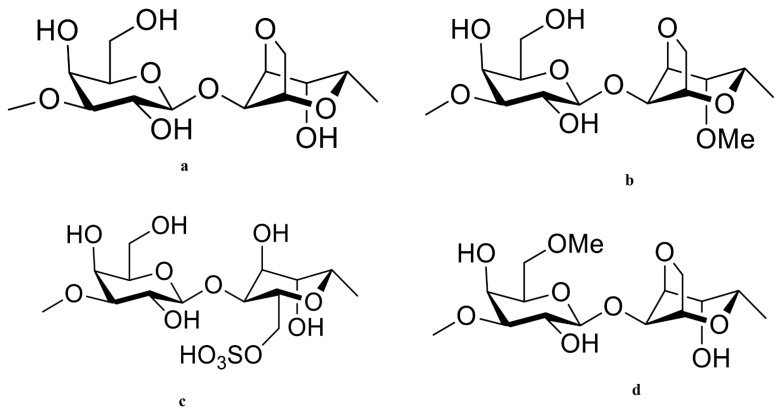
The Molecular structure of different types of porphyran with potential therapeutic effects are drawn in Chemdraw 12.0 Ultra. The Polymeric structures of porphyran such as G-A (**a**); G-A2M (**b**); G-L6S (**c**); and G6M-A (**d**).

**Red seaweeds:** Porphyran is a polymer found in *Porphyra* sp., a red seaweed. The porphyran is a galactose that has been heavily replaced by L-galactose 6-O-sulfation and 6-O-methylation [100]. The typical repetitive structure of porphyran is displayed (Figure 4). Porphyran is extracted from red seaweeds using hot water extraction, ultrasonic treatment, and radical degradation. Human studies have demonstrated the anticancer, hypolipidemic, and anti-inflammatory properties of porphyran [170]. When consumed orally, porphyran shields the livers of ICR mice from the effects of a high-fat diet, suggesting that it might be used as a dietary hypolipidemic component [171].

**Figure 5:**
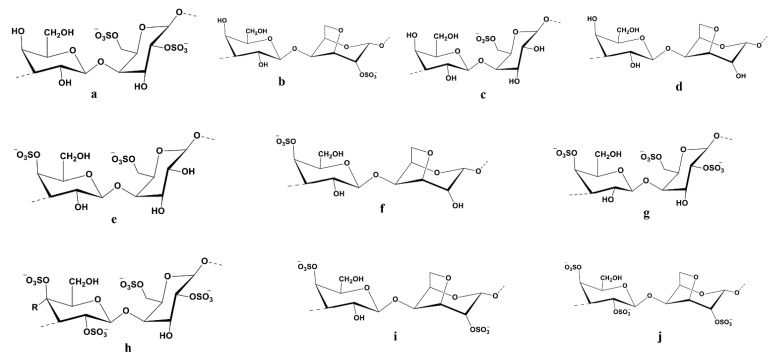
The Molecular structure of different types of carrageenan with potential therapeutic effects are drawn in Chemdraw 12.0 Ultra. The Polymeric structures of the different molecular structures of carrageenan as ϒ-carrageenan (**a**); β-carrageenan (**b**); δ-carrageenan (**c**); α-carrageenan (**d**); μ-carrageenan (**e**); κ-carrageenan (**f**); ν-carrageenan (**g**); ι-carrageenan (**h**); λ-carrageenan (**i**); and ϴ-carrageenan (**j**).

Carrageenan is one of the most common chemicals found in red algae. Carrageenan is a highly sulfated polymer found in the red algae family Rhodophyceae’s *Chondrus*, *Gigartina*, and various *Eucheuma* species. It is widely used as a gelling agent, stabilizer, binder, thickener, and additive in the food and pharmaceutical trades [47]. Carrageenan is a sulfated polygalactan with a virtual molecular mass of more than 100 kDa that includes 15 to 40% ester-sulfate. It is composed of α-1,3 and β-1,4-glycosidic links connecting substitute units of d-galactose and 3,6-anhydro-galactose (3,6-AG). Carrageenan is classified into numerous types, including κ, λ, ε, ι, μ and all of which contain 22 to 35 %, sulfate groups. The substance’s solubility in potassium chloride was utilized to classify it. The position and number of ester sulphate groups, as well as the amount of 3.6-AG, are critical factors in determining carrageenan-type properties. These terms refer to generic changes in the degree and composition of sulfation at certain locations in the polymer rather than specific chemical structures. Higher amounts of ester sulfate are associated with lower solubility temperature and gel strength. The ester sulfate percentage of kappa-type carrageenan ranges between 25 and 30%, and the 3,6-AG concentration is between 28 and 35%. The ester sulfate content of iota-type carrageenan ranges from 28 to 30%, while the 3,6-AG concentration ranges from 25 to 30%. Lambda-type carrageenan has an ester sulfate content ranging from 32 to 39%, with no 3,6-AG concentration [172]. Molecular structures of different carrageenan and their types are displayed (Figure 5). Apoptosis modulatory potential of a sulfated polysaccharide such as porphyran and carrageenan in cancer treatment is displayed (Table 3).

Cancer is known to be accelerated by free radicals and ROS (reactive oxygen species). Synthetic chemopreventive drugs usually generate undesirable side effects in the tumour environment due to their low selectivity and extensive biodistribution [173]. Porphyran is a potent chemopreventive agent due to its influence on cellular proliferation, the cell cycle, and the induction of apoptosis [174]. The red alga *Porphyra yezoensis* can induce apoptotic cell death in cancer cell lines in vitro while causing no cytotoxicity to normal cells. Generally speaking, porphyran is not toxic to healthy cells, but it is toxic to cancer cells, leading to dose-dependent cell death [175]. Additionally, it has been demonstrated that porphyran inhibits overall cell growth while inducing apoptosis in AGS human stomach cancer cells [175]. In AGS cells, the insulin-like growth factor-I receptor/Akt pathway increases PARP cleavage and caspase-3 activation, which encourages cell death [175]. Numerous studies have demonstrated the antitumor and anticancer properties of porphyran and its oligosaccharides. Porphyran can encourage the cleavage of poly (ADP-ribose) polymerase and the activation of caspase 3 in gastric cancer cells. By reducing the expression levels in AGS cells (gastric cancer), porphyran may slow the growth of cancer cells. This would then prevent IGF-IR phosphorylation and activate caspase 3 [175]. Crude and purified porphyran have antiproliferative activity in HT-29 and AGS cells *in vitro*. Apoptosis is induced by the crude porphyran polysaccharide component, as shown by an increase in caspase-3 activation [176]. Porphyran inhibits HT-29 cell proliferation by activating caspase-3 [176]. Porphyran has been shown to be effective against Ehrlich cells (EAC) carcinoma and Meth-A fibrosarcoma in mouse tumour models [177].

Porphyran-chungkookjang (made by 5% addition *w*/*w*) porphyran to fermented *Bacillus subtilis*, inhibited the proliferation of HT-29 and AGS cells more effectively than chungkookjang [178]. Porphyran inhibited cell proliferation and induced apoptosis in AGS cells, demonstrating clinical efficacy. Porphyran inhibits IGF-IR phosphorylation and activates caspase-3 [178]. A polysaccharide derived from *Porphyra yezoensis* was also found to inhibit the cancer cell cycle (G0/G1 or G2/M stages) [179]. Porphyran also reduces cell proliferation in the HeLa cells by inhibiting the cell cycle (G2/M phase) and altering the expression of cyclin B1, p21, p53, and CDK1 [45].

Natural porphyran was found to have no effect on MDA-MB-231, whereas two breakdown products had an impact when porphyran and two OPs (Oligo-porphyran) created by gamma irradiation were tested for anticancer activity. By preventing the cell cycle from entering into the G2/M phase, OPs have the capacity to reduce the growth of cells [45]. As a result, porphyran’s MW has displayed a significant impact on its anticancer efficacy. Low-MW of OPs are particularly effective against cancer; however, macromolecular porphyran has no antitumor activity. Furthermore, the anticancer activity of porphyran was discovered, with porphyran mainly acting as an anticancer drug by hindering cell growth and tempting apoptosis [180].

Carrageenans have been shown in numerous studies to have antiproliferative activity in cancer cell lines in vitro and tumour growth inhibitory effectiveness in mice [181,182,183]. They also have an antimetastatic effect by preventing cancer cells from connecting with the basement membrane and limiting tumour cell propagation and adhesion to different substrates; however, the precise mechanisms of action are yet unknown. Carrageenans from *Kappaphycus alvarezii* were found to prevent the growth of cancer cells from the liver, colon, breast, and osteosarcoma [184]. Yamamoto et al. (1986) discovered that taking various seaweeds orally significantly reduced the occurrence of carcinogenesis in vivo [185]. Hagiwara et al. (2001) [186] examined carrageenan’s effects on colonic carcinogenesis in male rats. Treatment had no effect on clinical symptoms or body weight. According to histological research, carrageenan has no colorectal carcinogenesis encouraging activity at the maximum dietary intake of 5.0% in the existing experimental settings [186].

Carrageenan has been shown in several studies to have specific cytotoxic effects on cancer cells. In such studies, doses of 250–2500 µg/mL of both k-carrageenan and λ-carrageenan inhibited human cervical cancer cells by stopping the cell cycle at specific stages and delaying its completion [47]. k-carrageenan delayed the cell cycle’s (G2/M stage), whereas λ-carrageenan delayed both the G1 and G2/M stages. However, k-selenocarrageenan (selenocarrageenan containing selenium) inhibits cell propagation in a human hepatoma cell. The cell cycle is terminated during the S phase of the cell cycle [187]. In vivo and in vitro studies, however, exposed that native carrageenan had no discernible anti-proliferation effect in the human osteosarcoma cell line. Because of a reduction in the Wnt/-catenin signalling pathway, degraded carrageenan-induced apoptosis inhibited tumour growth, and stopped the G1 phase of the cell cycle, all of which increased the existing rates of tumour-bearing mice [188].

Angiogenesis is a critical step in the progression of cancer. As a result, anti-angiogenic activity in cancer treatment is being extensively researched. Carrageenans are angiogenesis inhibitors due to their higher anti-angiogenic activity than suramin [189,190]. In the CAM model (chicken chorioallantoic membrane), the anti-angiogenic result of k-carrageenan oligosaccharides on ECV304 cells was demonstrated to limit cell proliferation, migration, and tube formation [191]. Furthermore, by negatively regulating human bFGFR, bFGF, CD105, and VEGF, oligosaccharides inhibited the formation of new blood vessels in MCF-7 xenograft tumours. Human umbilical vein endothelial cells were treated with λ-carrageenan oligosaccharides at relatively low concentrations (150–300 µg/mL), which had an adverse impact on the development of tumour blood vessel endothelial cells [192].

**Figure 6:**
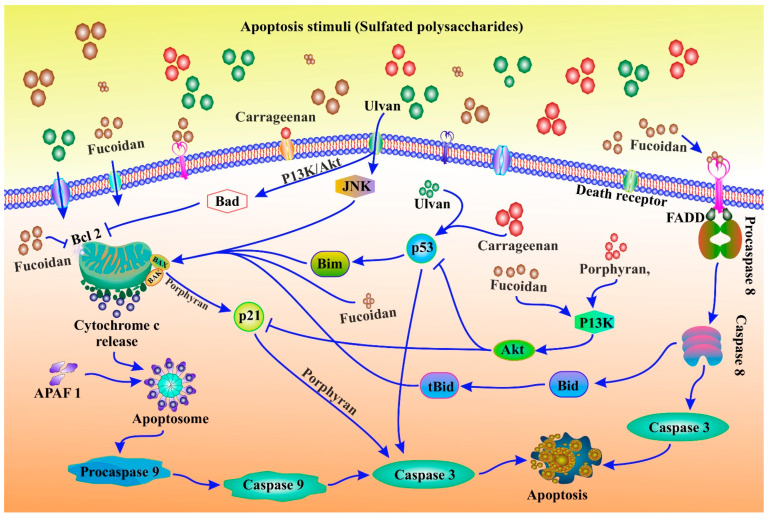
Apoptosis modulation by different sulfated polysaccharides derived from different seaweeds in cancer prevention. Ulvan induces JNK pathways that lead to mitochondrial ROS and displayed caspase-mediated apoptosis. Activation of caspase 8 via the death receptors FADD and fucoidan encourage extrinsic apoptosis. The expression of anti-apoptotic proteins like Bcl-2, Bcl-XL, and Mcl-1 is also downregulated by fucoidan. Additionally, fucoidan encourages the expression of proteins that aid in apoptosis, including Bax, Bid, and Bak. Induction of MOMP, translocation of Bax into the mitochondria, and release of cytochrome C from the cytosol promote intrinsic apoptosis. Fucoidan causes apoptotic cell death through the increased expression of caspase-3/7. Through the activation of procaspase 8 and caspase 8, which resulted in the cleavage of caspase 8, porphyran’s induce apoptosis through the modulation of death receptor-mediated apoptosis, caspase 3 activity, and caspase-dependent apoptotic cell death. Additionally, porphyran stimulated the mTOR/PI3K signalling pathways, which in turn activated the AKT signalling pathway and produced MDM2. This prevents P35 from activating caspase 3 and causing apoptosis. Porphyran also suppresses the expression of the anti-apoptotic proteins Bcl-xl and Bcl-2. They also increase Bax expression to promote apoptosis. Additionally, it triggers apoptosis and modulates intrinsic apoptosis by controlling the release of cytochrome C from the cytosol and the activation of caspase 9. Apoptosis is sparked by carrageenans, which also fight cancer. The appearance of the anti-apoptotic protein such as Bcl-xl, and Bcl-2 is downregulated by carrageenans. Carrageenans support intrinsic apoptosis by controlling the release of cytochrome C from the cytosol. For the purpose of causing apoptotic cell death, it induces the expression of caspase 3 and 9. In several cancer cell lines, carrageenans cause apoptosis by modulating caspase 3 activity through death receptor-mediated apoptotic cell death. Additionally, it prevents the cell cycle, which causes various cancer cells to undergo apoptosis.

**Figure 7:**
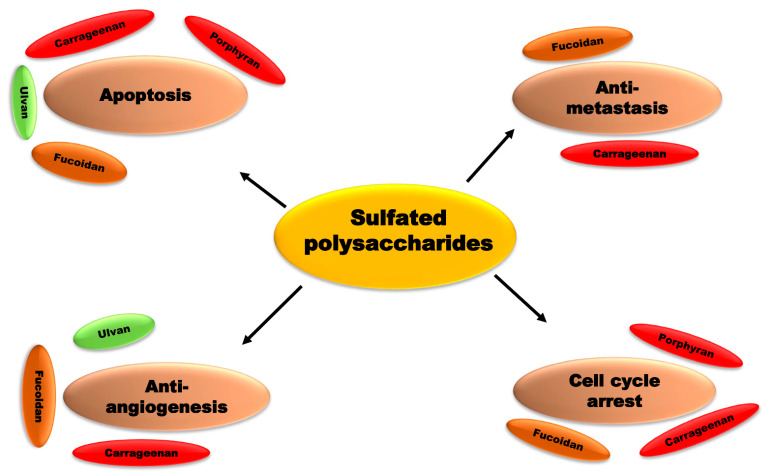
Sulfated polysaccharides displayed different chemopreventive roles in cancer. Sulfated polysaccharides such as ulvan, fucoidan, carrageenan, and porphyran displayed apoptosis in several cancer cell lines. Fucoidan, and carrageenan displayed potent antimetastatic effects in diverse cancer cell lines. Fucoidan, ulvan, and carrageenan displayed potent antiangiogenesis effects in different cancer cell lines. Moreover, fucoidan, carrageenan, and porphyran potential arrest cell cycles in cancer.

The amount and position of sulfation, as well as the molecular weight, influence the biological activity of sulfated polysaccharides. Chemical changes, in other words, alter the biological activities of carbohydrates [193]. For example, λ-carrageenan can be broken down into five different compounds with varying molecular weights, all of which have anti-cancer properties, most likely due to immunomodulation. Lower molecular weight products, such as those with molecular weights of 15 and 9.3 kDa, demonstrated superior anti-cancer and immunomodulatory properties [193]. Sulfation, acetylation, and phosphorylation improved the anti-cancer and immunomodulatory properties of k-carrageenan oligosaccharides from *Kappaphycus striatum*. Chemical modifications increased the oxidant activity of k-carrageenan oligosaccharides as well [194]. Sulfated polysaccharides from red algae and their apoptosis modulation in cancer therapeutics are displayed in Table 3. Induction of apoptosis is the mechanism adopted by chemopreventives. Different sulfated polysaccharides derived from different seaweeds trigger apoptosis in diverse cancer cell lines (Figure 6). Sulfated polysaccharides displayed different chemopreventive roles in cancer (Figure 7).

**Table 3:** Sulfated polysaccharides from red algae and their apoptosis modulating in cancer therapeutics.

Sl. No.	Name of the Sulfated Polysaccharides	Source of Sulfated Polysaccharides	Cell Line	Functional Involvement	Molecular Regulatory Pathways Involved	References
1	Porphyran	*Porphyra yezoensis*	AGS and HT-29	Appreciable inhibition of 54.7 % of tumour growth	-	[177]
2	Porphyran	*Ehrlich carcinoma*	Meth-A fibrosarcoma by intraperitoneal administration	Inhabited 58. 4%	-	[177]
3	Porphyran	*Ehrlich carcinoma*	SGC-7901 and 95D	Antiproliferation		[178]
4	Porphyran chungkookjang		AGS and HT-29	reductions in propagation (23–38%) of cancer cells	-	[178]
5	Porphyran	*Pyropia yezoensis*	Hep3B	Antiproliferation and cell cycle blocked in the G2/M phase	Upregulation of p21 and p53, while negatively regulation of cyclin B1 and CDK1	[45]
6	Porphyran	*Pyropia yezoensis*	HO-8910, MCF-7, K562, and SMMC-7721	inhibition of growth rates of cancer cells 21.2%, 23.6%, 19.8%, and 21% respectively	Antiproliferation and cell cycle arrested at the G0/G1or the G2/M check points	[179]
7	Crude porphyran	*Pyropia yezoensis*	HT-29 and AGS gastric	Antiproliferation and apoptosis-induced	Inclining caspase-3 activity	[176]
8	Crude porphyran	*Pyropia yezoensis*	HT-29 and AGS	Inhibition of cell growth in a dosé-dependent manner	Triggers apoptosis	[176]
9	Crude porphyran	*Pyropia yezoensis*	HT-29 and AGS	Inhibition by 50% of cancer cell growth	Initiation of apoptosis	[176]
10	purified porphyran	*Pyropia yezoensis*	HT-29 and AGS	Inhibition of cancer cell proliferation.	Initiation of apoptosis, as indicated by increased caspase-3 activity	[176]
11	Polysaccharide portion of the porphyran preparation	Commodity provided by Korea Bio Polymer (KBP) company	AGS	-	Negatively regulating IGF-IR phosphorylation and inducing caspase-3 activation	[175]
12	Polysaccharide	*Porphyra yezoensis*	HO-8910, MCF-7, K562, and SMMC-7721	Repressed the cancer cell cycle at the G_0_/G_1_ or G_2_/M stages	Initiation of apoptosis	[179]
13	Polysaccharide	*Porphyra yezoensis*	HeLa	decreases in cell proliferation by inhibition of the cell cycle at the G_2_/M phase	Altering the expression of p53, p21, cyclin B1, and CDK1	[45]
14	Oligo-porphyran	*Pyropia yezoensis*	HeLa	Inhibition of cell growth by prevention of the cell cycle from entering the G2/M phase		[45]
15	Porphyran	Purchased from Korea Bio Polymer (KBP)	AGS	Reduction of DNA synthesis and inhibition of cancer cell growth by both decreasing cell proliferation and inducing apoptosis.	Increase in poly (ADP-ribose) polymerase (PARP) cleavage, as well as the caspase-3 beginning, induces apoptosis	[175]
16	Porphyran	Purchased from Korea Bio Polymer (KBP)	AGS	Inhibition of cell growth and induction of apoptosis significantly	Induction of apoptosis via initiation of proapoptotic molecules, including Bax and caspase-3, and destruction of anti-apoptotic Bcl-2	[175]
17	Carrageenan	*Kappaphycus alvarezii*	Cancer cells derived from the liver, colon, breast, and osteosarcoma	Inhibition of the growth of cancer cells	-	[184]
18	λ-carrageenan and k-carrageenan	purchased	Human cervical cancer cells	-	Stops the specific stages of cell cycle and postpones the time of its completion	[47]
19	k-carrageenan		HepG2	Delayed the G2/M phase of the cell cycle	-	[187]
20	λ-carrageenan	purchased	HepG2	Delayed both the G1 and G2/M phases.	-	[187]
21	k-selenocarrageenan (k-carrageenan containing selenium)	purchased	HepG2	Displayed the anti-proliferative agent in the human hepatoma cell line	The cell cycle is terminated during the S phase	[187]
22	Degraded -carrageenan	purchased from Sigma	Human osteosarcoma	-	Repressed tumour growth, initiation of apoptosis, and halted the G1 phase, all of which improved the survival rates of tumour-bearing mice due to a decrease in the Wnt/β-catenin signalling pathway	[188]
23	k-carrageenan oligosaccharides	purchased from Changhang Colloid Technological Co., Ltd. (Jiangsu, China)	ECV304 cells in the chicken chorioallantoic membrane (CAM)	Displayed anti-angiogenic effect and limited cell proliferation, migration, and tube formation.	-	[191]
24	λ-carrageenan oligosaccharides		Human umbilical vein endothelial	Inhibition of the formation of new blood vessels in MCF-7 xenograft tumours by negatively regulating human VEGF, bFGF, bFGFR, and CD105.	-	[192]
25	λ-carrageenan oligosaccharides		Human umbilical vein endothelial	downregulation of intracellular matrix metalloproteinase (MMP-2) expression and had a negative effect on tumour blood vessel endothelial cell development	-	[192]

## 5. Nanoparticle Synthesis by Using Sulfated Polysaccharides and Its Impact on the Cancer Therapeutic Efficacy

The three main cancer treatments currently available are surgery, chemotherapy, and radiation therapy; chemotherapy, however, has not been the mainstay of cancer care in recent years due to the level to which it can harm healthy normal cells. Nanoparticles have emerged as alternative techniques for addressing only cancer cells, increasing the obtainability of drugs to cancer cells while sparing healthy cells from harm [195]. Seaweeds are a common source of natural sulfated polysaccharides, but there are other sources as well. Numerous biological and biomedical applications have been investigated for ulvan, carrageenan, porphyran, fucoidan, and their other derivatives in wound management, tissue engineering, drug delivery, and biosensors [196]. Seaweed polysaccharides interact with biological tissue readily because they have hydrophilic surface groups like carboxyl, hydroxyl, and sulphate [197].

Preparatory techniques that produce sulfated polysaccharide nanoparticles with the desired properties for efficient drug delivery systems have received a lot of attention [198,199]. Ionic gelation is typically a straightforward and gentle process for creating sulfated polysaccharide nanoparticles. However, to create ulvan, fucoidan, porphyran, and carrageenan-based nanoparticles with the desired shape, process optimization is crucial. The optimization can be carried out by adjusting the pH, temperature, concentration of calcium ions, concentration of sulfated polysaccharide, addition speed, and stirring rate. Both MCF7 and HepG2 cells are inhibited from proliferating by ulvan in nanoparticle albumin due to an increase in caspase-8 and caspase-9 levels, which denotes the induction of apoptosis [129]. When creating gold nanoparticles (AuNps), which are used as drug delivery systems for anticancer treatments, porphyran can also be used as a reducing agent. For instance, a human glioma cell line is more toxic to AuNps coated with porphyran (LN-229). As a result, porphyran-capped AuNps were developed and used as doxorubicin hydrochloride anticancer drug carriers [200]. A thymidylate synthase inhibitor called 5-fluorouracil (5-FU) has been used to treat cancer for a long time, but its use has been restricted because of side effects [201]. To create a water-soluble macromolecule for the prodrug 5-FU, porphyran-capped AuNps can be used as a drug carrier, delaying 5-FU release and minimizing side effects [202]. Porphyran-capped AuNPs were found to be safe in an in vitro cytotoxicity study, suggesting that they could be used as drug delivery systems [203]. Because of this, using porphyran as a reducing agent carrier for drug delivery has no unfavourable effects and might make it possible for anticancer medications to work more quickly. Fucoidan porphyran, and carrageenan-based nanoparticles in particular have been thoroughly investigated for the delivery of anti-cancer medications (Table 4).

## 6. Sulfated Polysaccharides Research Limitations and Future Expansion in Cancer Prevention

Although sulfated polysaccharides have numerous medicinal uses, their low bioavailability makes them impractical to use in daily life. Different sulfated polysaccharide structures affect how well they are absorbed in different organs [7]. Additionally, a continuous fluctuation in the effective doses in both in vitro and in vivo applications compromises their clinical trial [7]. The in vitro effectiveness of sulfated polysaccharides is frequently not replicated in preclinical or clinical studies [215]. Additionally, their sluggish intracellular metabolism and restricted solubility make a clinical application more challenging [216]. More significantly, their wide therapeutic application is a result of their cellular specificity and molecular target selectivity. Depending on the cellular, tissue, and tumour settings, these bioactive chemicals have different ways of causing cell death [216]. Additionally, clinical studies are more successful when the mono-specific and multi-specific functions of action are understood [216].

Synthetic analogues of sulfated polysaccharides might be more bioavailable, if they are created and tested [217]. To increase bioavailability and target specificity, sulfated polysaccharides and their synthetic equivalents may benefit from the use of micro-emulsions, nano-carriers, polymers, liposomes, and micelles [218]. These techniques, in our opinion, will be more frequently used in the future to create polysaccharide-based nanoparticles. In terms of delivering anti-cancer medications with increased bioavailability, seaweed polysaccharide-based nanoparticles have demonstrated promising results [170]. These techniques will also enhance their metabolism in host systems and solubility [170]. Additionally, the preclinical and clinical efficacy of apoptosis will be enhanced by its target specificity. Combining sulfated polysaccharides with drugs that have received FDA approval could significantly increase clinical effectiveness [170]. Additionally, sulfated polysaccharides, when added to or used as adjuvants in food, improve the therapeutic efficacy of modern medications [170].

## 7. Conclusions and Future Perspectives

The current cancer therapy system has identified sulfated polysaccharides as a trustworthy source for discovering bioactive druggable molecules with a variety of chemotherapeutic effects in various malignancies. Over half of the FDA-approved medications in recent years have been directly extracted from marine sources or created using a chemical counterpart. The isolation and use of these sulfated compounds from marine sources have greater bioavailability, diversified chemical makeup, and non-reductant cytotoxicity. Owing to these characteristics, the seaweed-derived sulfated polysaccharides act as possible lead pharmacophores in treating various malignancies. However, a significant barrier to their pharmaceutical utilization is their bioavailability, improved separation, cleanliness of the isolates, and target selectivity as one drug multi-target specificity and cell/tissue/cancer context. Additionally, they play a significant role as druggable mediators due to their wide variety of therapeutic interventions, low-cost commercial production, and promising pre-clinical and clinical applications. Meanwhile, there is some optimism for commercializing these sulfated polysaccharides from marine seaweeds due to the extensive on- and off-site harvesting of the organisms and low-cost cultivation upkeep. Additionally, the large-scale manufacture of these sulfated polysaccharides for chemotherapy is made more effective by the out-of-range application of chemical synthesis of these polysaccharides. With the advent of new prospects for the isolation and screening of sulfated polysaccharides from seaweed as innovative pharmacological agents against various cancers, the chemotherapeutic use of such prospective agents is likely to flourish in the near future. Moreover, nanoparticles mediated sulfated polysaccharide-based nanoparticles are capable of sustained drug release, high stability, and biocompatibility, all of which will care their use in clinical trials in the future. Targeting moieties will increase the therapeutic efficacy of polysaccharide-based nanoparticles while minimizing undesirable side effects. Additionally, creating such drug candidates will improve currently available medications for the advancement of personalized and precision medicine.

## Figures and Tables

**Table 1:** The physicochemical characteristics of sulfated polysaccharides and their therapeutic potential with other functions.

Sl. No.	Sulphated Polysaccharide	Source	Physiochemical Properties	Therapeutic Use	Other Applications
1	Ulvan	*Ulva rigida*, *Ulva pertusa*, *Ulva compressa*, *Ulva intestinalis, Ulva prolifera*, *Ulva lactuca Ulva pertusa*, *Ulva conglobata*, *and Epiactis prolifera*	Viscosity, sulfate content, molecular weight, metal ions, rheological property.	Antioxidant, anti-inflammatory, anticancer, antibacterial, antiviral, immunomodulating, antihyperlipidemic, anticoagulant and tissue engineering	Food, agriculture
2	Fucoidan	*Laminaria japonica*, *Saccharina japonica*, *Undaria pinnatifida*, *Dictyopteris* spp., *Ecklonia cava, Ascophyllum nodosum*, *Cladosiphon okamuranus*, *Fucus vesiculosus*, *Fucus evanescens*, *Dictyota menstrualis*, *Sargassum polycystum*, *Dictyota delicatula*, *Turbinaria conoides*, *Saccharina latissimi*, *Spatoglossum asperum*, *Cystoseira sedoides*, *Coccophora langsdorfii*	water-soluble, Ionic crosslinking, Solubility, nontoxic, viscous, biocompatibility, and biodegradability	Anticancer, antidiabetic, Alzheimer, Parkinson, cardiovascular, antiviral, immunomodulatory, antibacterial, wound healing, antiaging, antifungal, anticoagulant, anti-inflammatory, antioxidant, antiviral and antitumor activity	Food, cosmeceutical, preserving moisture, removing freckles, fertilizer
3	Porphyran	*Porphyra haitanensis*, *Porphyra yezoensis Porphyra tenera*, *and Ehrlich carcinoma*	Molecular weight, intrinsic viscosity, bioavailability and bioactivity adhesion, the position of sulfate groups, degree of sulfation, sugar type and glycosidic bond and chain conformation	Antioxidant, anticancer, wound healing, anticoagulant, antihypertensive, Immunomodulation	Addition of nori, food
4	Carrageenan	*Kappaphycus alvarezii* and *Eucheuma denticulatum.*	Rheological properties, viscosity, viscoelasticity, water-soluble polymers, gelling, stabilizing, and viscosity-building agent.	Antioxidant, Anticancer, Antitumor, anticoagulant and antithrombotic properties, Immunomodulatory, anti-inflammatory,	Cosmetics, emulsifiers, stabilisers, colloids, or gum, flavoured in milk, newborn formulae, nutrition supplements, dairy products, beverages, hand lotion, shampoos, pharmaceutical industries, drug formation, wound dressing, agriculture, painting, household products, and industrial effluents. food applications such as dairy products, jellies, pet foods, and sauces.

**Table 2:** Sulfated polysaccharides from brown algae as potent anticancer agents. ↑: upregulation, ↓: Downregulation, ⊥: Inhibition.

Sl. No	Name of Sulfated Polysaccharide	Sulfated Polysaccharides Sources	Cell Line Involved	Cancer Types	Practical Participation	References
1	Fucoidan	*Cladosiphon navae-caledoniae Kylin*	MDA-MB231 and MCF-7	Breast	↓ Bcl-xl, Bcl-2, and Mcl-1	[46]
2	Fucoidan	*Sargassum hemiphyllum*	MCF-7	Human breast	↓ miR-29c and miR-17-5p, PI3K/Akt pathway inactivation	[138]
3	Fucoidan	*Sargassum* sp.	Lung carcinoma cells (LCC) line and melanoma B16	Lungs and melanoma	⊥ cell proliferation	[137]
4	Fucoidan	*Fucus vesiculosus*	MDA-MB231	Breast	↓ Bcl-2, Bcl-xl, and Mcl-1	[46]
5	Fucoidan	*Laminaria gurjanovae*	JB6 Cl41	*In vivo* model	EGFR regulation and Induction of c-jun signalling by EGF and blocking of activator protein-1 (AP-1)	[139]
6	Fucoidan	*Fucus vesiculosus*	ES-2 and OV-90	Ovarian	Declined proliferation, arrested cell cycle, the release of cytochrome c, generation of ROS and ER stress via PI3K and MAPK signalling inactivation cascades	[140]
7	Fucoidan	*Fucus vesiculosus*	MC3	Human muco-epidermoid carcinoma	p-38 MAPK, ERK1/2, and JNK pathway regulation	[140]
8	Fucoidan	*Fucus vesiculosus*	HeLa G-63, Hep G2,	Liver carcinoma	⊥ cell proliferation	[142]
9	Fucoidan	*Fucus vesiculosus*	HCT-15	Colon	pro-caspase-9, pro-caspase-3 induction and ↓ Bcl-2	[144]
10	Fucoidan	*Fucus vesiculosus*	HT-29, HCT116	Human colon	Caspases-8, -9, -7, and -3 induction, and cleaved PARP levels. Bak, Mcl-1, Bid	[144]
11	Fucoidan	*Cladosiphon novaecaledoniae*	MCF-7, HeLa, and HT1080	Breast, Cervical and Colon	Caspase-8 induction and -9, ROS generation, MAPK, MEK1, PI3K/Akt, MEKK1, ERK1/2 and JNK signalling regulation	[147]
12	Fucoidan	*Fucus vesiculosus*	HS-Sultan	Lymphoma	activation of Caspase-3 and ERK and GSK pathways regulation	[143]
13	Fucoidan	*Fucus vesiculosus*	HL60	Human caucasian promyelocytic leukaemia	MEKK1, ERK1/2, MEK1, and JNK	[146]
14	Fucoidan	*Cladosiphon novaecaledoniae* Kylin	MCF-7, MDA-MB-231 and HeLa, and HT1080	Breast and Cervical	Displayed Intrinsic apoptosis pathways, nuclear condensation, DNA fragmentation, and ↓ Bcl-2	[147]
15	Fucoidan	*Cladosiphon okamuranus*	HL60	Human caucasian promyelocytic leukaemia	Displayed intrinsic apoptosis through caspase-3/7	[145]
16	Fucoidan	*Cladosiphon okamuranus*	U937	Lymphoma	Caspase-3 and -7 induction and displayed caspase-dependent apoptotic signalling pathway	[148]
17	Fucoidan	*Cladosiphon okamuranus*	MCF-7	Breast	Caspase-7, caspase-8, caspase-9 induction	[151]
18	Fucoidan	*Undaria pinnatifida*	PC-3	Prostate	ERK1/2 MAPK p38 MAPK regulation (PI3K)/Akt signalling pathway, Wnt/β-catenin signalling pathway regulation	[155]
19	Fucoidan	*Fucus vesiculosus*	MCF-7	Breast	EMT process regulation	[152]
20	Fucoidan	*Cladosiphon okamuranus*	Colon 26-bearing mouse model	Colon in vivo model	Tumour growth suppression	[154]
21	Fucoidan	*Cladosiphon okamuranus*	MCF-7	Breast	Displayed caspase-independent cell death pathways	[152]
22	Fucoidan	*Cladosiphon okamuranus*	MCF-7	Breast	Condensation of chromatin material, internucleosomal DNA fragmentation and induction of caspase-7, caspase-8, caspase-9	[151]
23	Fucoidan	*Undaria pinnatifida*	MG-63 and metastatic breast cancer	Osteosarcoma and breast	cellular blabbing, nuclear fragmentation and chromatin condensation	[167]
24	Fucoidan	*Fucus vesiculosus*	MCF-7	Breast	Regulation of cell proliferation by cyclin D1 and CDK-4	[160,161]
25	Fucoidan	*Fucus vesiculosus*	MCF-7	Breast	↓ MMP-9 and ↑ E-cadherin	[162]
26	Fucoidan	*Undaria pinnatifida*	A549, HepG2, HeLa and PC-3	Lung, Cervical, Liver and Prostate	Activation of caspase-8 and -9, and ROS generation, PI3K/Akt, MAPK, MEK1, MEKK1, ERK1/2 and JNK signalling regulation	[156]
27	Fucoidan	*Undaria pinnatifida*	DU-145	Prostate	MAPK and p-PI3K/PI3K/p-Akt/Akt regulation p-ERK and p-P38 signalling and ↑ Bax expression	[166]
28	Fucoidan	*Fucus vesiculosus*	MC3	Human muco-epidermoid carcinoma	p-38 MAPK, ERK1/2, and JNK pathway	[140]
29	Fucoidan	*Undaria pinnatifida*	MG-63	Osteosarcoma	Membrane blabbing, nuclear fragmentation and chromatin condensation to induce apoptosis	[167].
30	Fucoidan	*Undaria pinnatifida*	leukaemia	Leukemia	p38 MAPK activation and Bcl-2 modulation	[167].
31	Fucoidan	*Fucus vesiculosus*	4T1 and MDA-MB-231	Lung	TGFR/Smad/Snail, Slug, Twist and EMT axes regulation	[163]
32	Fucoidan	Synthetic fucoidan	human bladder cancer EJ cells	Human bladder	Induced G1 cell cycle arrest through modulation of cyclin E, cyclin D1, and cyclin-dependent-kinases (Cdks)	[159]
33	Fucoidan	Synthetic fucoidan	5637 cells	Human bladder	↑ Bax/Bcl-2 and PI3K/Akt signalling regulation	[168]
34	Fucoidan	Synthetic fucoidan	5637 and T-24	urinary	↓ MMP-9 mediated by ↓ NF-kB and AP-1	[169]
35	Fucoidan	Synthetic fucoidans	MDS/AML and SKM-1	Myelodysplastic syndromes (MDS)	PI3K/Aktsignallingg regulation	[165]
36	Fucoidan	Synthetic fucoidan	5637 cells	Urinary bladder	MMP, enhanced Bax/Bcl-2 ratio and cytosolic release of cytochrome C.	[168]

**Table 4:** Nanoparticle synthesis by using sulfated polysaccharides and its impact on the cancer therapeutic efficacy.

Sl. No.	Materials Used	Preparation Methods	Particle Size in nm	Drugs Used	References
1	Chitosan–fucoidan NPs	Self-assembled	100	PLL	[204]
2	O-carboxymethyl chitosan/fucoidan	Ionic cross-linking	270	Curcumin	[111]
3	Fucoidan NPs	Self-assembly	140	Doxorubicin	[205]
4	Chitosan–fucoidan	Ionic gelation	173	Curcumin	[206]
5	Carrageenan/protamine	Self-assembled	100–150	NA	[207]
6	Chitosan/carrageenan	Ionic complexation	350–650	Ovalbumin	[208]
7	Chitosan/carrageenan/TPP	Ionic gelation	150–300	BSA	[209,210]
8	Carrageenan hydrogel	Gelation	NA	Methylene blue	[211]
9	Chitosan–carrageenan NPs	Ionotropic gelation	200 to 1000	rHu-EPO	[212]
10	Carboxymethyl chitosan and carrageenan	-	-	Riboflavin	[213]
11	Cross-linked–carrageenan NPs	Reverse microemulsion	100	Methylene blue	[214]

## Data Availability

Not applicable.

## Abbreviations

bFGF, basic fibroblast growth factor; BAX, Bcl-2-associated X protein; Bcl-2, B-cell lymphoma 2; Bcl-xl, B-cell lymphoma-extra-large; HIF-1, Hypoxia Inducible Factor-1; IL2, interleukin-2; JNK, c-Jun N-terminal kinase; PP1 and PP2, protein phosphatase 1 and 2; ROS, reactive oxygen species; TNF, tumor necrosis factor; Bim, BCL-2–interacting mediator of cell death; APAF 1, Apoptotic protease activating factor-1; PI3K, phosphatidylinositol-3 kinase; FADD, Fas-associated death domain protein.
